# Systematic Review of Auditory Training Outcomes in Adult Cochlear Implant Recipients and Meta-Analysis of Outcomes

**DOI:** 10.3390/jcm13020400

**Published:** 2024-01-11

**Authors:** James R. Dornhoffer, Shreya Chidarala, Terral Patel, Karl R. Khandalavala, Shaun A. Nguyen, Kara C. Schvartz-Leyzac, Judy R. Dubno, Matthew L. Carlson, Aaron C. Moberly, Theodore R. McRackan

**Affiliations:** 1 Department of Otolaryngology-Head and Neck Surgery, Mayo Clinic, Rochester, NY 55905, USA; dornhoffer.james@mayo.edu (J.R.D.); khandalavala.karl@mayo.edu (K.R.K.); carlson.matthew@mayo.edu (M.L.C.); 2 College of Medicine, University of Florida College of Medicine, Gainesville, FL 32610, USA; shreya.chidarala@ufl.edu; 3 Department of Otolaryngology-Head and Neck Surgery, University of Pittsburgh Medical Center, Pittsburgh, PA 15260, USA; patelt5@upmc.edu; 4 Department of Otolaryngology-Head and Neck Surgery, Medical University of South Carolina, Charleston, SC 29425, USA; nguyensh@musc.edu (S.A.N.); leyzac@musc.edu (K.C.S.-L.); dubnojr@musc.edu (J.R.D.); 5 Department of Otolaryngology-Head and Neck Surgery, Vanderbilt University Medical Center, Nashville, TN 37212, USA; aaron.c.moberly@vumc.org

**Keywords:** cochlear implant, aural rehabilitation, auditory training

## Abstract

**Objective:** to review evidence on the efficacy of auditory training in adult cochlear implant recipients. **Data Sources:** PRISMA guidelines for a systematic review of the literature were followed. PubMed, Scopus, and CINAHL databases were queried on 29 June 2023 for terms involving cochlear implantation and auditory training. Studies were limited to the English language and adult patient populations. **Study Selection:** Three authors independently reviewed publications for inclusion in the review based on a priori inclusion and exclusion criteria. Inclusion criteria encompassed adult cochlear implant populations, an analysis of clinician- or patient-directed auditory training, and an analysis of one or more measures of speech recognition and/or patient-reported outcome. Exclusion criteria included studies with only pediatric implant populations, music or localization training in isolation, and single-sample case studies. **Data Extraction:** The data were collected regarding study design, patient population, auditory training modality, auditory training timing, speech outcomes, and data on the durability of outcomes. A quality assessment of the literature was performed using a quality metric adapted from the Grading of Recommendations Assessment, Development, and Evaluation (GRADE) Working Group guidelines. **Data Synthesis and Meta-Analysis:** Data were qualitatively summarized for 23 studies. All but four studies demonstrated significant improvement in at least one measured or patient-reported outcome measure with training. For 11 studies with sufficient data reporting, pre-intervention and post-intervention pooled means of different outcome measures were compared for 132 patients using meta-analysis. Patient-direct training was associated with significant improvement in vowel-phoneme recognition and speech recognition in noise (*p* < 0.05 and *p* < 0.001, respectively), and clinician-directed training showed significant improvement in sentence recognition in noise (*p* < 0.001). **Conclusions:** The literature on auditory training for adult cochlear implant recipients is limited and heterogeneous, including a small number of studies with limited levels of evidence and external validity. However, the current evidence suggests that auditory training can improve speech recognition in adult cochlear implant recipients.

## 1. Introduction

Cochlear implantation has become the standard of care for rehabilitation of moderate to profound sensorineural hearing loss, with studies showing consistent improvement in speech recognition and quality of life following implantation [1,2]. Such successes have spurred the gradual broadening of cochlear implant (CI) candidacy criteria, with the global economic impact of cochlear implantation expected to exceed USD 2.5 billion in the next several years [3,4,5]. Despite advances in device design, surgical technique, and programming strategies, post-CI speech recognition still falls well short of normal hearing, with significant variability in speech recognition outcomes [6,7,8]. Unfortunately, other than changes in CI programming by audiologists, few interventions are available to help improve CI outcomes following surgery. One potential avenue to optimize CI outcomes is aural rehabilitation. Broadly, comprehensive aural rehabilitation consists of the components of sensory management, instruction, counseling, and perceptual training, with the latter most often being auditory (or audiovisual) training [9]. However, while likely beneficial to the CI population, current evidence to guide these interventions remains scarce. To address this knowledge gap, this systematic review and meta-analysis summarizes the available evidence regarding the efficacy of auditory training in adult CI users [10,11,12,13,14,15]. 

Hearing with a CI is a unique experience compared to normal acoustic hearing. While patients typically show significant improvements in speech recognition and quality of life, there remains a deficit in speech recognition, with mean word recognition ability in the quiet of approximately 50–70% and substantial unexplained variability among individuals [6,8,16]. A large portion of hearing deficits and variability among patients may be related to patients having to learn how to process the electrically coded speech signal. For some patients, this process comes passively during daily life, but for others, it may require more intentional practice or rehabilitation. For some adults, the learning process for adaptation to hearing with a CI can be protracted, with reports of peak CI speech recognition ability reaching 1–2 years after implantation [17,18]. Post-CI auditory training may improve or accelerate this learning process and is inexpensive or free, in contrast to the high costs of cochlear implantation itself. 

A variety of auditory training exercises have been developed, but there is no standardization for use in the adult CI population [10]. In general, auditory training can be broken down into two categories: patient-directed, at-home exercises and clinician-directed training sessions, which are typically led by a speech-language pathologist. The patient-directed, at-home exercises include passive listening exercises, such as listening to recorded speech or audiobooks, listening to the radio, or watching television, and active listening exercises. Active exercises can include listening exercises with communication partners with feedback, speech-tracking exercises, or computer-based auditory training (CBAT), which employs interactive software that has often been developed by CI companies. 

Research on the effectiveness of auditory training in adult CI users is scarce, but the limited data show evidence of speech and quality-of-life benefits in those patients who pursue auditory training; a recent survey of CI audiologists demonstrated that 85% recommended the use of such resources in the immediate post-activation period [10,14,15,19,20]. A report by Dornhoffer et al. also showed benefits in CI-specific quality-of-life outcomes with the use of at-home CBAT software programs [21]. However, many extant studies are limited in their scope and external validity, fail to include commonly available forms of auditory training, or have an insufficient sample size to draw meaningful conclusions [10,11,12,13,14,15]. As a result, most audiologists and physicians are limited to empirically recommending various patient-driven rehabilitation strategies due to scant evidence on the benefits of specific resources [10,11,12,13]. Therefore, a formal evaluation of the efficacy of commonly used clinician- or patient-directed auditory training techniques is of great importance to fill this research gap. 

This study aims to provide an updated literature review and meta-analysis to determine the effectiveness of commonly available auditory training/listening activities in improving outcomes related to speech recognition and CI-specific quality of life. The goal is to provide preliminary, specific recommendations on appropriate post-CI auditory training, which can offer an affordable measure by which to optimize implant outcomes. 

## 2. Material and Methods

### 2.1. Systematic Search Strategy

The literature search was performed following the Preferred Reporting Items for Systemic Reviews and Meta-analyses (PRISMA) guidelines [22]. Inclusion criteria were generated using the Participants, Intervention, Control, Outcomes, and Study Designs (PICOS) strategy. PICOS inclusion and exclusion criteria are detailed in Table 1 and broadly include studies examining post-CI auditory training in adult patients [23]. This study was not registered.

### 2.2. Study Identification

**Figure 1:**
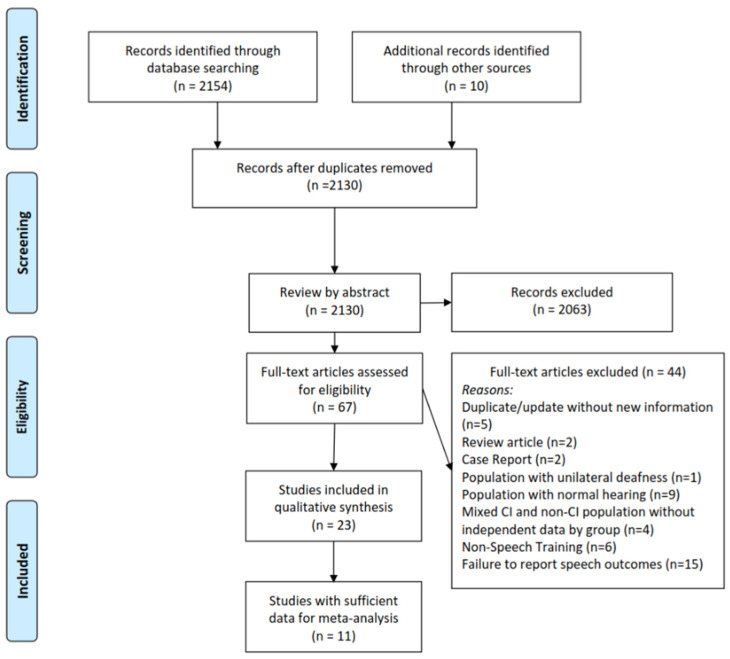
Flow diagram of study selection, eligibility, and inclusion.

A flow diagram of the study identification and review is detailed in Figure 1. Three reviewers independently searched the PubMed, Scopus, and CINAHL databases on 29 June 2023 for appropriate studies of auditory training for adult CI recipients. The following search terms were used: cochlear implant OR cochlear implantation OR cochlear implants AND listening activities OR aural rehabilitation OR aural training OR training OR activities OR exercises OR auditory training OR auditory rehabilitation OR speech therapy OR speech pathology OR speech pathologist. The following filters were employed: English language, full text, and adult patient. Our search yielded 2154 articles. Ten additional articles were identified from a previous literature review by Cambridge et al. and an analysis of review article reference lists encountered in our search [24]. After removing duplicate articles, 2130 unique articles were identified. 

### 2.3. Study Screening and Selection

Articles identified in our search were reviewed by title and abstract for our PICOS inclusion and exclusion criteria by three independent reviewers (J.R.D., T.P., and K.R.K.). Discrepancies were resolved by a senior author. There was no time range or limitation on the publication date. After review by title and abstract, 67 articles underwent full-text review for inclusion. After a full-text review, 23 articles were included in the review. Reasons for dismissal of full-text articles from review included: duplicate article or update of article without new information (*n* = 5), review article (*n* = 2), case report (*n* = 2), population with unilateral deafness (*n* = 1), population with normal hearing (*n* = 9), mixed CI and non-CI population without independent data by group (*n* = 4), non-speech training (*n* = 6), and no report of speech recognition outcomes (*n* = 15). Additionally, articles were reviewed to ensure no overlapping study populations were included. 

### 2.4. Data Extraction

Data collected from studies included the first author, year of publication, study design, patient population, patient demographics, implant laterality, implant duration of use, target pre-intervention implant performance if noted, training modality, training frequency, training duration, training time period, training environment, speech recognition scores, patient-reported outcome scores, reports of auditory training compliance where available, and any commentary offering insight on follow-up outcome measures or the durability of outcome measures (e.g., data collection any period of time after the initial post-training timepoint). If a study had two study arms or protocols without crossover and with different populations, each population and its data were selected and detailed separately.

Given the heterogeneity of the interventions noted in the literature, different interventions were grouped based on the level of clinician interaction: patient-directed, which were interventions solely reliant on patient usage of the resource; clinician-directed, which were interventions that relied heavily on clinician interaction or guidance during a patient’s use of the training program; and mixed, for those interventions with distinct portions performed independently by the patient and also with clinician interaction.

### 2.5. Analysis of Quality

Assessment of quality was performed utilizing a metric adapted from that utilized by Henshaw et al. for the analysis of auditory training [25]. The metric was developed in accordance with the Grading of Recommendations Assessment, Development, and Evaluation (GRADE) Working Group guidelines [26]. Three authors (J.R.D., T.P., and K.R.K.) independently reviewed each article and assigned their quality assessment. If unanimity was not achieved, scores were assigned to the majority score or averaged if all authors disagreed.

The metric involved assessing ten factors for quality in each study. Five factors dealt with general scientific quality: randomization, presence of a control, power calculation/large sample size, blinding, and scientific reporting of outcome measures. Low scores on such factors would indicate poor internal validity or the potential for bias. Five factors dealt with the general external validity of each study with respect to real-world auditory training: external validity of outcome measure, external validity of training or training environment, reporting on training compliance, reporting of long-term or durable results (outcome reporting any time after the initial post-intervention period), and the presence of constructive/corrective feedback during training. Scores for each factor ranged from 0 to 2. As per Henshaw et al., a score of 0 indicated flawed or no information from which to make a judgment, a score of 1 indicated weak information or lack of detail, and a score of 2 indicated appropriate use and reporting [25]. Scores were totaled for each factor to form an overall quality assessment score. As adapted from the GRADE guidelines, studies with scores from 0 to 5 represented a very low level of evidence, 6 to 10 a low level of evidence, 11 to 15 a moderate level of evidence, and 16 to 20 a high level of evidence.

### 2.6. Statistical Analysis

Studies with sufficient reporting on outcomes and population data were included in a meta-analysis of outcomes. Each study’s sample data were combined, with the weighted mean and standard deviation determined. Differences were noted using the variable delta (Δ). Pre-intervention and post-intervention pooled means of each subdomain were compared using a comparison of weighted means using a meta-analysis of continuous measures performed with Cochrane Review Manager (RevMan Version 5.4 Cochrane Collaboration 2020). The fixed-effects model was used after consideration of both fixed- and random-effects models. This assumption is tested using the heterogeneity test or I^2^ statistic. I^2^ values of 25%, 50%, and 75% represented low, medium, and high heterogeneity, respectively. If this test yields a low probability value (*p* < 0.05), then there is a high likelihood that the fixed-effects model is invalid and the random-effects model is more appropriate [27]. The random-effects model incorporates both the random variation within the studies and the variation between the different studies. 

## 3. Results

### 3.1. Study Characteristics

The study characteristics are summarized in Table 2. In total, 23 publications were identified that met a priori inclusion and exclusion criteria. Publication dates ranged from 1991 to 2023. One study by Tyler et al. featured two different treatments for different populations. As such, these were considered as separate studies [28]. The majority of studies (*n* = 13) were repeated measure studies, examining a single uncontrolled cohort [13,20,28,29,30,31,32,33,34,35,36,37,38]. A total of seven studies were randomized controlled trials or crossover trials [39,40,41,42,43,44,45], and four studies were nonrandomized controlled trials or cohort studies [21,28,46,47].

### 3.2. Patient Populations

The characteristics of the study samples are detailed in Table 2. With the exception of Dornhoffer et al., an observational study of 72 patients [21], patient samples were generally small (*n* = 3–24), with the next largest experimental group being 24 CI recipients [41]. Most studies (*n* = 13) examined a majority of patients with unilateral implantation, with only Tyler et al. reporting bilaterally implanted patients [28]. 

Most studies examined patient populations of experienced CI users. Only four studies examined auditory training in new CI users [21,31,43,47]. Of those studies examining experienced CI recipients, nine targeted specific levels of pre-training CI performance. Fu et al. [20], Ingvalson et al. [33], and Borel et al. [30] reported on patients with poor word recognition scores, subjective poor performance, or poor performance with a phone, respectively. In contrast, Schuman et al. [39], Barlow et al. [29], and Greene et al. [29] included CI users with high pre-training word or speech recognition. All other studies did not report on the population targeted for the intervention or examined typically performing CI users. 

### 3.3. Interventions

All studies utilized unique resources or algorithms for auditory training. Most studies utilized or reported on some form of CBAT. Six studies utilized the Computer Assisted Speech Training (CAST) program developed at the House Ear Institute [20] or programs developed from it, such as Angel Sound™ [20,21,34,35,38,47]. All other studies employed custom CBAT programs created for the individual study or featured interventions still in development. 

A small number of studies (*n* = 4) did not utilize any form of computer program-based intervention. Gagne et al. [31] utilized an individualized, clinician-based auditory training strategy; Bernstein et al. [45] also reported on a clinician-based strategy combined with equipment and listening-strategy counseling. Ihler et al. [44] utilized a CD recording of spectrally filtered or normal speech in various conditions and difficulties, the degree of which was chosen by patient preference, and Borel et al. [30] utilized listening tasks directed by a speech-language therapist over a phone. Dornhoffer et al. [21] reported on patients utilizing clinician-directed therapies and passive-home-based exercises; however, patients were free to use any resource, such as a CBAT program. Moberly et al. [47] and Völter et al. [37] reported on a strategy using both clinician-directed and computer-based training. Specific details of the training exercises used in each study are described in Table 3. 

Most studies exclusively used some form of phoneme-, word-, or digit-recognition exercise in various signal-to-noise ratios (SNR) as training material. A smaller proportion utilized additional or alternative training methodologies. Tyler et al. used localization training in addition to word recognition training [28]. Ihler et al. used spectrally filtered speech to train CI use with a phone [44]. Borel et al. [30] used clinician-directed listening exercises over the phone [30]. Lastly, Shafiro et al. used environmental noise identification exercises, which included human vocalization in addition to common environmental sounds [36]. Given the current study’s focus on speech recognition skills, studies with music and localization training in isolation were excluded from our analysis based on our a priori criteria.

The length, timing, and duration of interventions varied widely among studies. The length of individual training sessions, when prescribed, ranged from fifteen minutes to three hours. Most studies utilized daily, or close to daily, training, although three studies employed a twice-weekly intervention and one used weekly training [31,39,42,46]. Two studies used a combination of weekly clinician-directed training with additional training either based on patient preference [47] or completed daily [37]. The overall duration of the studies ranged from 4 days to 16 weeks, with the majority of studies offering training over the course of 3–6 weeks. 

Most studies offered auditory training at home, with nine requiring at least one portion of intervention in a laboratory or clinic setting. However, home-based interventions did not always represent a normal home environment. Six studies with patient-directed training in the home actively monitored compliance, with notifications from the research team for patients to resume or complete their auditory training [13,29,32,35,38,44]. Additionally, Tyler et al. [28] required patients to use specific speakers and a unique speaker setup that was provided by the lab for their home-based intervention.

### 3.4. Qualitative Analysis of Outcomes 

A summary of CI outcome measures for each study is detailed in Table 4. As per our a priori inclusion criteria, included studies were required to report at least one measure of speech recognition or patient-reported outcome measure. Beyond that, there was no one measure that was common to all studies examined, with studies employing variable outcome measures in a variety of noise conditions. Less than half of studies used patient-reported outcome measures [13,21,30,34,37,41,43,44,45,47]. As with speech measures, patient-reported outcomes varied, with no single instrument common to all studies. Examples of patient-reported outcome measures utilized included the Cochlear Implant Quality-of-Life 35 Profile [48], the Abbreviated Profile of Hearing Aid Benefit (APHAB) [49], the Glascow Benefit Inventory [50], the Hearing Handicap Inventory [51], and the Nijmegen Cochlear Implant Questionnaire [52].

A summary of outcomes demonstrating statistical significance for each study is also detailed in Table 4. Despite the large variety of training stimuli and outcome measures, all but four studies demonstrated significant improvement in at least one measure of phoneme, word, or sentence recognition with training [31,36,42,47]. Where reported, improvements were often generalizable, with a majority of the studies demonstrating improvement in off-task outcome tests, meaning word/sentence stimuli used in the outcome measure were not included in the trained stimuli. For example, Ihler et al. [44] trained CI users with spectrally filtered speech in order to use phones more proficiently, but the cohort that trained with the spectrally filtered speech also had improvement in tests of unfiltered speech in quiet as compared to the control group. Additionally, Miller et al. (2008) [42] showed that patients trained in their Speech Perception and Training System (SPATS) improved in recognition of both study materials and untrained CNC (Consonant-Nucleus-Consonant) lists and HINT (Hearing in Noise Test) sentences in quiet and in noise. However, this was not universal, as Reis et al. [41] reported little to no off-task training in a randomized cross-over study of a CBAT protocol with and without visual components. For studies detailing at least one patient-reported outcome measure, significant improvement was reported for most [21,30,37,41,43,45], but not all studies [13,34,44,47]. No study showed a decline in any patient-reported outcome measure with training. Similarly, no study showed a significant decline in any measure of speech recognition.

Regarding the durability of these improvements, 13 studies reported on follow-up or data taken after the cessation of training. The time period in these studies ranged anywhere from 4 days to 8 months. Most studies that reported follow-up showed the durability of the training effect. Green et al. [32], for example, showed that the SRTs of Bamford–Kowal–Bench (BKB) [53] and IEEE sentences in both male and female voices remained stable or even continued to improve over the 1-month post-training period. Schumann et al. [39] also showed stability of training at 6 months for speech recognition in noise at 0 dB SNR and +5 dB SNR. However, Reis et al. [41] demonstrated that significant improvements were seen in CNC word scores in quiet and the Quality-of-Life Scale [54]. Scores immediately after training were no longer significant relative to baseline one to three months after the cessation of training.

### 3.5. Meta-Analysis of Outcomes

**Figure 2:**
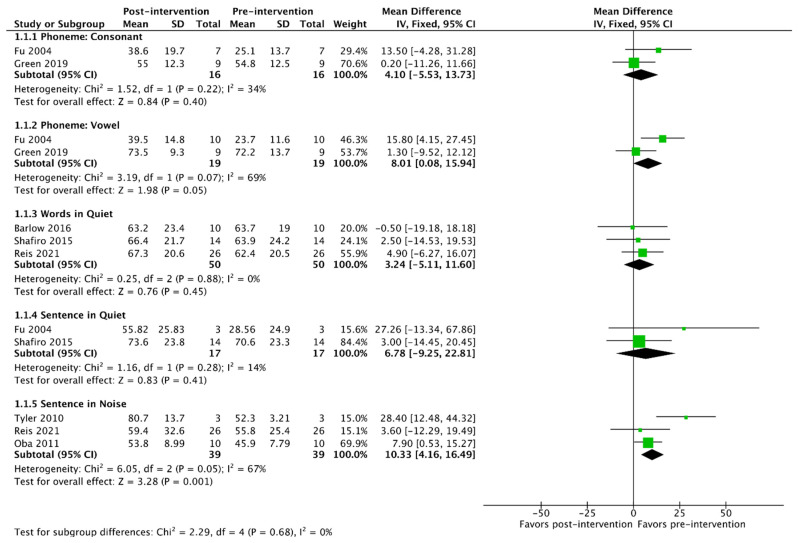
Meta-analysis of outcomes for patient-directed interventions [20,28,29,32,35,36,41].

**Figure 3:**
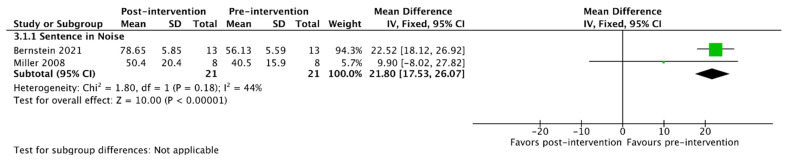
Meta-analysis of outcomes for clinician-directed interventions [42,45].

**Figure 4:**
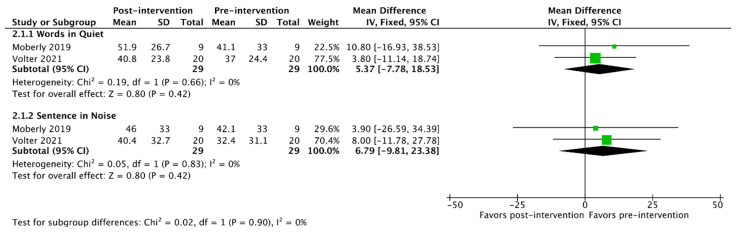
Meta-analysis of outcomes for mixed: patient-directed and clinician-directed interventions [37,47].

Of the 23 studies included in this review, 11 had sufficient data reporting for meta-analysis of outcomes, as detailed in Table 4. A summary of the pooled patient sample for meta-analysis is detailed in Table 5. For the purposes of outcome synthesis, each meta-analysis was performed based on the type of auditory training provided. The pooled analysis outcomes are detailed for each type of training in Table 6 and in Figure 2, Figure 3 and Figure 4. The heterogeneity of outcomes ranged from low to medium in this body of the literature.

Of the studies included in the meta-analysis, seven detailed the use of some form of primarily patient-directed training intervention. Table 6 and Figure 2 display that there was a significant improvement in vowel phoneme recognition in quiet and sentence recognition in noise. No other improvements were observed. 

Considering interventions that were primarily clinician-directed, two studies were identified. The only common metric of speech recognition available for meta-analysis was sentence recognition in noise, which demonstrated a significant improvement from pre- to post-intervention (Table 6 and Figure 3). 

Finally, two studies were identified that utilized a mixed intervention with distinct clinician-directed and patient-directed components. Word recognition in quiet and sentence recognition were available for pooled analysis in these studies (Table 6 and Figure 4). While both metrics trended toward improved outcomes from pre- to post-intervention, the change was not significant (95% confidence interval of improvement crosses 0). 

### 3.6. Quality Assessment

The total quality assessment scores and scores for each quality assessment factor are detailed in Table 7. Overall, the quality of the literature on this topic is low. Only eight studies qualified as a moderate level of evidence, which is the highest seen in this body of the literature. The remaining 15 studies had either a very low level of evidence (2) or a low level of evidence (13). 

## 4. Discussion

### 4.1. Overview

Learning to listen with a new CI is often equated to learning a new language. To help with this process, auditory training is often recommended for new CI recipients. These training approaches may range from simple at-home exercises, such as listening to an audiobook or radio, or they may involve focused, in-office therapy with a clinician or therapist. Computer-based CI training programs have also been developed, with each major CI company having developed its own proprietary software in addition to several other programs that are free or available for purchase. Angel Sound^TM^ is one program developed in part from the CAST and SoundExpress computer programs used by Fu et al. [20], Oba et al. [35], and Zhang et al. [38]. Unfortunately, while auditory training is almost universally recommended, there is a paucity of the literature on the subject among adult CI users, and there is a lack of consensus on recommendations to guide auditory training or broader aural rehabilitation programs. In this study, we have reviewed the extant literature and found evidence of improvements in CI outcomes with auditory training, but studies generally suffer from low-quality evidence.

### 4.2. Efficacy of Auditory Training

Twenty-three studies were ultimately reviewed to analyze the effect of auditory training on CI outcomes. Most examined the effects of some form of patient-directed CBAT (Table 3), with a handful analyzing clinician-directed training or a combination of clinician-directed and/or other patient-directed interventions such as listening to spectrally filtered recorded stimuli on a CD [21,30,31,37,44,45,47]. Outcome measures varied widely from study to study and ranged from validated metrics, such as CNC words or AzBio sentences, to study-specific measures of speech recognition and sound localization. Of the 23 studies, 9 included at least one patient-reported outcome measure [13,21,30,34,37,41,43,44,47]. However, outcome measures were heterogeneous, and outcome reporting was variable, limiting the inclusion of some studies in the meta-analysis. 

Meta-analysis of the available studies demonstrates an overall benefit from different types of auditory training, with significant improvements in various measures of speech recognition for both clinician-directed and patient-directed interventions (Figure 2, Figure 3 and Figure 4). However, while general benefit is demonstrated, the effectiveness of any specific training resource cannot be ascertained from this review due to near-universal small sample sizes and oftentimes mixed results within individual studies, with patients improving significantly on some measures while failing to improve on a similar measure in the same sample (Table 3). Additionally, the effectiveness of training—the ability to have a meaningful impact on patients under typical clinical conditions—is unclear based on the more controlled assessment and training settings applied for most of the studies reviewed.

Similarly, few conclusions can be drawn regarding the durability of outcomes. Only half of the studies collected data at any time beyond the immediate testing period. However, where available, all follow-up data appear largely to show durable outcomes, excepting one study that showed that improvement in quality of life was no longer significant 3 months after training [41].

Given the sparse literature on the efficacy of auditory training, other systematic reviews to date have been limited. Sweetow et al. [55] performed a similar systematic review on the efficacy of auditory training for non-CI hearing-impaired patients, and Henshaw et al. [25] reviewed the literature on auditory training in the general population, including CI and non-CI patients. Both reviews were limited by the number and quality of publications at their respective times of authorship; however, both reported similar results to this review. Namely, the evidence is heterogeneous and limited in statistical power, but the literature generally supports auditory training as a possible therapy for hearing-impaired patients. These conclusions are given with particular note to the affordability and lack of risk entailed in most auditory training exercises. Cambridge et al. [24] reviewed the results of auditory training in adult CI users after 2010. Their findings are similar to our own, albeit with a limited number of studies and a lack of meta-analyses, likely secondary to a limited search window. That said, as with the current study, they showed that all studies demonstrated some benefit in at least one measure of speech recognition skill, but the quality of the data was limited, concluding that only two studies controlled for covariables in such a manner that benefits could be attributable to training effects alone [39,46]. Rayes et al. [56] published a review of auditory training for pediatric CI users. Despite different target age groups, their findings are similar to our own in adults, namely, auditory training appears to afford benefits in both trained and off-task measures of speech recognition. However, as in the current review, the quality of the data and reporting of off-task testing were limited. Additionally, no studies on pediatric CI auditory training, as described by Rayes et al. [56], reported on patient-reported outcome measures.

While we do see that the literature on auditory training generally demonstrates benefits, we can primarily comment on CBAT, as this is the training modality utilized in the vast majority of studies. Only one study [31] examined clinician-directed therapy in isolation and did so with only four patients and no statistical analysis beyond qualitative assessment. Clinician-directed therapy was examined in three additional studies but in combination with other training modalities [21,47]. Similarly, only one study [44] examined a patient-directed training modality that was not a computer program using a CD of various speech scenarios. As such, the conservative findings that we derive from this review are primarily applicable to CBAT. The efficacy of many common exercises recommended to CI recipients, such as listening to an audiobook or the radio, is less certain. That said, longitudinal data from the use of such exercises in newly implanted adults demonstrated no significant benefits of these activities at 3 months post-activation [21].

### 4.3. Quality of Literature

While the outcomes of auditory training can be generalized as beneficial, the ability to make firm conclusions is restricted. The studies we examined were generally limited to one or two domains: the internal validity of the study and the external validity of the auditory training. Regarding internal validity, studies were often inadequate with respect to sample size, utilization of control groups, blinding, and randomization. As such, the power of these studies is limited. Moreover, the common lack of a control group makes it difficult to parse out the effect of intervention from passive CI learning, particularly in less experienced CI users. 

Regarding external validity, we found that most studies failed to directly address the modalities of auditory training that are available for the standard CI recipient. As detailed above, almost all studies examined study-specific computer programs or programs still under development, and only six studies use a resource that is widely available or was ultimately developed into a currently available CBAT program (e.g., Angel Sound^TM^) [20,21,34,35,38,47]. Therefore, the results of many of these studies are not necessarily generalizable to the CBAT that is typically recommended by clinicians for adult CI users. Additionally, all but four studies included experienced CI users. The greatest gain in speech understanding for an average CI recipient is in the first 3 months, with some additional gain occurring up to 2 years [8,57]. Therefore, the application of these training interventions in the early post-CI period is likely important, as this is a key period of learning and plasticity for CI recipients. Unfortunately, the timing of many studies in the current literature precluded any evaluation of the value of auditory training during this time period. 

### 4.4. Limitations and Future Goals

The limitations of this study were primarily related to the availability and quality of published data, as detailed above. Available data were generally heterogeneous and often not in compliance with recent guidelines for outcome reporting; as such, meta-analysis was only possible using data from less than half of the identified studies [58]. Additionally, due to a scarcity of data, meta-analysis was not possible on patient-reported outcome measures. Therefore, we can offer mostly qualitative generalizations regarding the impact of auditory training on the functional abilities of adult CI users. In general, auditory training appears to result in improved speech recognition in experienced CI users, but the strength of these effects and their generalizability to the overall adult CI population are low. 

Future research will require additional prospective analysis of larger CI samples to determine the effect of commonly available exercises and interventions for auditory training on CI recipients. Studies should be designed to determine the efficacy of specific interventions that are widely available for use in the clinical setting. Future studies should also assess the impact of the timing of auditory training on CI outcomes, particularly during periods of rapid speech recognition growth, such as the early post-CI period. The current literature deals primarily with experienced CI users and often fails to assess the impact of auditory training during the key 6–12 months of CI speech recognition gain immediately after device activation [8].

## 5. Conclusions

Auditory training for CI users appears to be beneficial in improving various measures of speech recognition and quality-of-life. However, the extant literature is markedly variable in training modality, outcome measure, and quality of reporting. These limitations impede making definitive conclusions regarding the efficacy of any specific form of auditory training. Interventions in the literature are also often nonrepresentative of clinically available forms of auditory training. As such, future prospective studies are necessary to optimize post-CI auditory training. However, given their low cost and risk, practitioners can offer general recommendations for auditory training in that both clinician- and patient-directed approaches appear to provide benefits for adult CI users.

## Figures and Tables

**Table 1:** PICOS inclusion criteria.

Participant	Cochlear implant recipient, implanted as an adult (≥18 years)
Intervention	Any clinician- or self-directed auditory training (excluding music or localization training)
Control	Studies were required to compare post-training outcome measures against pre-training outcomes in a repeated-measures manner or against outcomes from an untrained control cohort
Outcome	One or more measures of speech recognition or patient-reported functional or quality-of-life outcomes
Study	Randomized controlled trials, non-randomized controlled trials, cohort studies with control groups, and repeated measure studies (excluding single-sample case studies)

**Table 2:** Details of study design and patient populations.

Aural Rehab							
First Author (Year)	Study Design	Sample Size (Control)	Age Mean (Range)	Gender (% Female)	UL/BL	CI Experience	Performance Status at Baseline
Barlow (2016) [29]	RM	10 (NA)	55 (39–72)	NR	100% UL	>15 months	High performance (WRS > 50%)
Bernstein (2021) [45]	RCT	13 (12)	Trained: 66.2 (48–80) Control: 62.8 (47–85)	Trained: 69% Control: 67%	100% UL	>3 months Range: 0.25–1.3 years	Target speech recognition between 10 and 85% correct
Borel (2020) [30]	RM	9 (NA)	71 (46–82)	89%	NR	NR	Poor performance with phone†
Dornhoffer (2022) [21]	CC	CDT: 13 (59) PHT: 42 (40) CBAT: 24 (48)	69 (NR)	46%	100% UL	Newly implanted	NR
Fu (2004) [20]	RM	10 (NA)	43 (25–60)	60%	100% UL	>12 months	Low performance (WRS < 50%)
Gagne (1991) [31]	RM	4 (NA)	40 (27–64)	50%	100% UL	Newly implanted	NR
Green (2019) [32]	RM	9 (NA)	61 (48–70)	NR	NR	>10 months Avg. 2.4 years	High performance (minimum 80% BKB sentences)
Ihler (2017) [44]	RCT	10 (10)	Trained: 59 (NR) Control: 55 (NR)	Trained: 40% Control: 90%	100% UL	>6 months Avg 1.8 years	Normal performance (WRS > 40%, <90%)
Ingvalson (2013) [33]	RM	5 (NA)	71 (50–85)	60%	80% UL	>1 year Range: 2–14 years	Subjective poor performance
Kerneis (2023) [34]	RM	15 (NA)	51 (18–69)	54%	67% UL	>1 year Range: 1–12 years	NR
Magits (2023) [43]	RCT	20 (20)	Trained: 63 (NR) Control: 63 (NR)	Trained: 55% Control: 55%	Trained: 95% UL Control: 90% UL	25% New implantees 75% Experienced Range: 0.1–15.9 years	NR
Miller (2008) [42]	RCT	8 (8)	Trained: 58 (35–81) Control: 53 (42–79)	NR	NR	>1 year Range: 1–19 years	NR
Miller (2016) [46]	Non-RCT	9 (5)	61 (46–75)	71%	31% UL	>6 months Range: 0.7–23 years	Normal performance
Moberly (2020) [47]	Non-RCT	6 (control 7; active control 7)	Trained: 68 (55–77) Control: 65 (49–91) Active control: 68 (54–76)	Trained: 17% Control: 57% Active control: 33%	95% UL	New implantees	NR
Oba (2011) [35]	RM	10 (NA)	66 (46–78)	60%	NR	>1 year Range: 1–20 years	NR
Reis (2021) [41]	RCS	24 (24)	63 (42–84)	NR	69% UL	Minimum 1 year; 1–25 years	NR
Reynard (2022) [40]	RCT	15 (15)	Trained: 48 (24–76) Control: 60 (45–75)	Trained: 53% Control: 47%	NR	>1 year Range: 1–26 years	NR
Schumann (2015) [39]	RCT	15 (12)	Trained: 60 (49–75) Control: 61 (34–76)	Trained: 73% Control: 58%	Trained: 53% UL Control: 75% UL	>2 years Avg. trained: 4.2 years Avg. control: 4.6 years	High performance
Shafiro (2015) [36]	RM	14 (NA)	63 (51–87)	64%	NR	>1 year Avg. 5 years	NR
Stacey (2010) [13]	RM	11 (NA)	55 (23–71)	45%	NR	>3 years Range 3–11 years	NR
Tyler a (2010) [28]	RM	3 (NA)	60 (43–63)	66%	BL	>3 years Range: 3–8 years	NR
Tyler b (2010) [28]	Non-RCT	3 (6)	Trained: 69 (63–77) Control: NR	Trained: 66% Control: 50%	100% BL	Minimum 5 years; range: 5–8 years	NR
Völter (2021) [37]	RM	20 (NA)	59 (26–82)	70%	NR	>3 months Range: 3–22 months	NR
Zhang (2012) [38]	RM	7 (NA)	64 (71–78)	71%	100% UL *	>2 years Avg. 4.3 years	NR

RM = repeat measures, RCT = randomized controlled trial, Non-RCT = non-randomized controlled trial, RCS = randomized crossover study, CC = controlled cohort, UL = unilateral, BL = bilateral, CDT = clinician-directed training, CBAT = computer-based auditory training, PHT = passive home-based training, NA = not applicable, NR = not reported, and WRS = word recognition score. * With hearing aid in contralateral ear, †did not use CI with phone.

**Table 3:** Details of study interventions.

Author (Year)	Training Category	Specific Training Details	Frequency	Length of Individual Sessions	Duration of Total Intervention	Location	Compliance
Barlow (2016) [29]	Patient-directed	Custom computer program: psychophysical task training, including gap-in-noise detection, frequency discrimination, spectral-rippled noise, iterated noise, and temporal modulation	Daily	1 h	7 days	Home	Compliance enforced by protocol
Bernstein (2021) [45]	Clinician-directed	Combination of a custom auditory training program consisting of vowel–consonant contrast, sentence identification, and speech-tracking exercises along with informational and communication strategy counseling	Weekly	90 min	6 weeks	Lab	Compliance enforced by protocol
Borel (2020) [30]	Clinician-directed	Custom telephone training program: progressive listening tasks through phone conducted remotely with a speech therapist	3/week	15–25 min	6 weeks	Home	Compliance enforced by protocol
Dornhoffer (2022) [21]	Mixed	Self-directed training: no training, clinician-directed training, passive-home-based training, or CBAT	NA	NA	NA	Home	Compliance not reported
Fu (2004) [20]	Patient-directed	Computer-Assisted Speech Training (CAST) program: phonemic recognition training; tailored to baseline performance	5/week	1 h	>1 month	Home	Compliance not reported
Gagne (1991) [31]	Clinician-directed	Clinician-directed phoneme and sentence recognition exercises directed by patient preference and baseline	Weekly	3 h	12 weeks	Lab	Compliance enforced by protocol
Green (2019) [32]	Patient-directed	Custom computer program: word identification training from male and female recordings of English phrases in 20 talker babble with foils to the chosen word	Daily	30 min	4 weeks	Home	Compliance: 96.2/96 planned sets
Ihler (2017) [44]	Patient-directed	Heidelberg Training CD (2 groups: spectrally filtered to mimic phone signals and normal) CD includes recordings of spoken word lists, poems, and recorded short stories; sorted by difficulty, which is increased by patient preference	Daily	15 min	10–14 weeks	Home	Avg daily time: Filtered: 19.5 ± 16.5 min Non-filtered: 16.4 ± 8 min
Ingvalson (2013) [33]	Clinician-directed	Seeing and Hearing Speech program: vowel and consonant identification in words, sentences, and phrases with multitalker babble played in background with level varied to patient performance	Daily	1 h	4 days	Lab	Compliance not reported
Kerneis (2023) [34]	Patient-directed	French version of Angelsound™: access only to phonemic contrast training	5/week	30 min	4 weeks	Home	Avg 31.7 ± 4 min/day Avg 10.3 ± 1.7 total hours
Magits (2023) [43]	Patient-directed	CBAT program–Leuven Interactive Scheme for Hearing Training Evaluation and Audiological Rehabilitation (LUISTER) controlled against a training program using same training materials but not personalized on performance	5/week	15–20 min	16 weeks	Home	Majority exceeded goal hours
Miller (2008) [42]	Clinician-directed	Speech perception assessment and training system (SPATS): syllable onset, nuclei recognition, and sentence recognition in quiet and in babble	2/week	2 h	6 weeks	Lab	Compliance enforced by protocol
Miller (2016) [46]	Patient-directed	Custom computer program: phoneme recognition training with multiple different voices with more voice options added with patient performance	2/week	2 h	2 weeks	Lab	Compliance enforced by protocol
Moberly (2020) [47]	Mixed	Comprehensive auditory rehabilitation (CAR) program including individualized clinician-directed therapy with audiology and speech pathology, a one-hour preoperative counseling session, and self-directed home-based training with Angel Sound™; active control consisted of standard of care with a one-hour preoperative counseling session	Weekly CBAT: daily	1 h 30 min	8 weeks	Home and lab	Compliance reported for overall study with 79.2% of original enrollees completing the study
Oba (2011) [35]	Patient-directed	SoundExpress (based on CAST program, now part of Angel Sound): digits-in-noise training	5/week	30 min	4 weeks	Home	Avg time: 647/600 min planned
Reis (2021) [41]	Patient-directed	Two training programs used in crossover fashion: a custom CBAT program using IEEE sentences and Maryland CNC words with four-talker babble presented at random with close-set stimulus identification and a computer-based visual training program that used a similar format to the prior but with a partially obstructed visual representation of the stimulus presented simultaneously with auditory stimulus	5/week	13 min for nonvisual training; 9.5 min for visual training	6 weeks	Home	71% completing non-visual training; 58% training visual training
Reynard (2022) [40]	Patient-directed	Custom, serious game training program with progressive training activities and SNR	>20 sessions	NA	5 weeks	Home and lab	Avg time: 13 h
Schumann (2015) [39]	Patient-directed	Custom computer program: vowel consonant group (VCV and CVC) recognition supervised by clinician	2/week	45–60 min	3 weeks	Lab	NA
Shafiro (2015) [36]	Patient-directed	Custom computer program: environmental sound identification tailored to patient performance on pretesting	4/week	40–60 min	1 week	Home	NA
Stacey (2010) [13]	Patient-directed	Custom computer program: alternative forced choice word identification training and sentence training using IEEE and low-context SPIN sentences	5/week	1 h	3 weeks	Home	8/11 participants completing 15 h
Tyler a (2010) [28]	Patient-directed	Custom computer program with 8 speakers: localization training and Spondee word in babble recognition training	Daily	>30 min	1–3 months	Home	NA
Tyler b (2010) [28]	Patient-directed	Custom computer program with 2 mobile speakers: localization training and Spondee word in babble recognition training	Daily	>30 min	1–3 months	Home	NA
Völter (2021) [37]	Mixed	Clinician directed training followed by training with the CBAT program Train2Hear	CDT: weekly CBAT: NA	2 h	6 weeks *	Home and lab	CDT: enforced by protocol CBAT: NA
Zhang (2012) [38]	Patient-directed	SoundExpress (based on CAST program, now part of Angel Sound): phoneme contrast training (vowels and consonants) for 6 of 7 subjects and monosyllabic word identification in babble for 1	5/week	30 min	4 weeks	Home	Avg: 18/20 goal hours

CDT = clinician-directed training, CBAT = computer-based auditory training, IEEE = Institute of Electrical and Electronics Engineers, and NA = not applicable. * Total of 3 weeks of clinician-directed and 3 weeks of CBAT.

**Table 4:** Speech outcomes, statistical significance, and long-term follow-up for included studies.

Author Year	Speech Outcome Measure(s)	Results	Statistical Significance	Durability of Outcomes	Data Sufficient for Meta-Analysis
Barlow (2016) [29]	Lexical Neighborhood Test in Quiet	No improvement from 64 (19)% to 63 (23.4)%	No	No long-term follow-up	Yes
	Lexical Neighborhood Test in Noise	Improvement from 36 (21)% to 47 (22)%	Yes **		
Bernstein (2021) [45]	CasperSent sentence recognition test	Significant improvement in trained cohort at 1 week (mean improvement of 19% correct) and 2 months (improvement of 22.52% correct)	Yes, for pre- and post-training analysis *** Yes, for comparison to control *	2 months	Yes
	Speech tracking	Improvement of 24.13 words per minute in the trained group	Yes **		
	Glascow Benefit Inventory	Greater benefit in trained group at 1 week and 2 months	Yes *		
	Hearing Handicap Inventory	Reduction in mean handicap score by 26.15 of 100 at 1 week and 27.13 of 100 at 2 months	Yes, for pre- and post-training analysis *** Yes, for comparison to control *		
	Nijmegen CI Questionnaire	Improvement all domains for both groups	Yes, for pre- and post-training analysis for both trained and control groups * No, for comparison to control		
	Client-Oriented Scale of Improvement	Significant improvement in all goals at 1 week and 2 months	Yes, for pre- and post-training analysis *** Yes, for comparison to control *		
Borel (2020) [30]	Lafon words with direct voice	No improvement (−10%)	No	1 month	No
	Lafon words with recorded voice	No improvement (−1%)	No		
	Lafon words via phone	Improvement by 13%	No		
	MBAA sentences with direct voice	Improvement by 8%	No		
	MBAA sentences with recorded voice	Improvement by 17%	Yes ***		
	MBAA sentences via phone	Improvement by 13%	Yes *		
	MBAA sentences with recorded voice in noise +5SNR	No improvement (0%)	No		
	Self-assessment of ease with phone use	Improvement by 28 out of 100	Yes ***		
	Self-assessment of self-confidence with phone use	Improvement of 3 out of 10	Yes **		
	Self-assessment of stress with phone use	Reduction of 2 out of 10	No		
	Number of phone calls using implant	Increase of 11	Yes ***		
Dornhoffer (2022) [21]	CNC phoneme in quiet	Improvement in CBAT users by 33% after multivariable regression compared to control; no effects from other training types	Yes *	3 months	No
	CNC word in quiet	Improvement in CBAT users by 23% after multivariable regression compared to control; no effects from other training types	No		
	AzBio sentences in quiet	Improvement in CBAT users by 33% after multivariable regression compared to control; no effects from other training types	Yes *		
	Cochlear Implant Quality-of-Life 35 Profile Score	Improvement in CBAT users by 10.9 points out of 100 in global score, 13.9 points out of 100 for the communication domain, and 19.5 points out of 100 for the entertainment domain after multivariable regression compared to control; improvement in CDT users by 19.8 points out of 100 for the social domain; and no effects from other training types	Yes *		
Fu (2004) [20]	Consonant recognition	Improvement from 25 to 38%	Yes **	No long-term follow-up	Yes
	Vowel recognition	Improvement from 22 to 36%	Yes ***		
	HINT sentences (only 3 subjects tested)	Improvement from 28 to 56%	Yes **		
Gagne (1991) [31]	Sentence Understanding Without Context Test	1 subject improved in sentence understanding	No	No long-term follow-up	No
	Continuous discourse tracking	All subjects improved in discourse tracking	No		
Green (2019) [32]	SRT BKB sentences male voice (dB SNR)	Improvement from 5.5 to 4.1; 3.4 at follow-up	Yes ***	1 month	Yes
	SRT BKB sentences female voice (dB SNR)	Improvement from 2.9 to 2.2; 1.5 at follow-up	Yes ***		
	IEEE sentences male voice (dB SNR)	Improvement from 8.2 to 6.3; 6.9 at follow-up	Yes ***		
	IEEE sentences female voice (dB SNR)	Improvement from 8.0 to 5.4; 4.6 at follow-up	Yes ***		
	Vowel identification	No improvement	No		
	Consonant identification	No improvement	No		
Ihler (2017) [44]	Sentence recognition in quiet with spectral filtering to mimic phones (Modified Oldenburg Sentence Test)	Improvement from 70 to 79% for filtered; 71 to 74% for nonfiltered	Yes, for filtered CD group only *	No long-term follow-up	No
	Word discrimination in quiet (Freiburg Monosyllabic Test)	Improvement from 56 to 69% for filtered; no improvement from 66 to 65 for nonfiltered	No		
	Abbreviated profile of hearing aid benefit	Improvement from 40 to 35 for filtered; no improvement from 36 to 40 in nonfiltered	No		
Ingvalson (2013) [33]	HINT sentences	Improvement in HINT quiet and HINT +15; no score listed in text	Yes **	4 days	No
	Quick SIN	Improvement in quick SIN; no score listed in text	Yes *		
Kerneis (2023) [34]	Vowel recognition in quiet	Improvement at post-training and 1 month follow-up	Yes ***	1 month	No
	Consonant recognition in quiet	Improvement at post-training and 1 month follow-up	Yes ***		
	French HINT sentences (dB SNR)	SNR lower at post-training and 1 month follow-up	No statistical analysis		
	Speech, Sound, and Quality-12 questionnaire	No improvement	No		
Magits (2023) [43]	Digit-in noise-testing, which was on task for training material	Improvement at post-training for both arms without difference between arms	Yes ***	8 months	No
	Phoneme identification testing, which was on task for training material	Improvement at post-training for both arms without difference between arms	Yes ***		
	Leuven Intelligibility Sentences Test in sound field (dB SNR)	Improvement by 2 dB SNR for experimental arm and 1.5 dB for control; changes durable at 8 months	No		
	Nijmegen CI Questionnaire	Improvement at post-training for both arms at post-training; scores not significantly different between post-training and at 8 months	Yes **		
Miller (2008) [42]	SPATS Test–onset, nucleus, and sentence recognition in quiet	6% change trained, 0% untrained	No	No long-term follow-up	No
	SPATS Test–onset, nucleus, and sentence recognition in noise	14% change trained, 2% change untrained	No		
	CNC word in quiet	6% change trained, −5% change untrained	No		
	HINT quiet	13% change trained, −7% change untrained	No		
	HINT +10 SNR	10% change trained, 1% change untrained	No		
Miller (2016) [46]	Phoneme identification	Improvement in test group by 11.5%; no improvement in control	Yes (post hoc testing significant improvement only in da-ba discrimination) **	No long-term follow-up	Yes
Moberly (2020) [47]	AzBio sentences in quiet	More rapid improvement in experimental and active control arms at 3 and 6 months post-activation (71.5% and 80.9%, respectively, vs. 70.5% for control at 6 months)	No	No long-term follow-up	Yes
	AzBio sentences in 10-talker babble	More rapid improvement in experimental and active control arms at 3 and 6 months post-activation (45% and 52.8%, respectively, vs. 43.8% for control at 6 months)	No		
	CNC words	No differences between arms	No		
	Nijmegen CI Questionnaire	No differences between arms	No		
	Hearing Handicap Inventory for Adults/Elderly	No differences between arms	No		
	Speech, Spatial, and Qualities of Hearing Scale	No differences between arms	No		
Oba (2011) [35]	Digit SRT in steady noise and babble	Improved from −2.2 to −5.0 dB SNR in steady noise; improved from 2.7 to −1.3 dB SNR in babble	Yes, in both conditions **	1 month	Yes
	HINT sentence recognition in steady noise and babble	No improvement in steady tone (6.6 to 5.6 dB SNR); improved from 11.2 to 8.3 dB SNR in babble	Yes, only in babble *		
	IEEE sentences–moderate SNR in steady noise and babble	Improved from 59 to 66% correct in steady noise; improved from 33 to 42% correct in babble	Yes, in both conditions **		
	IEEE sentences–difficult SNR in steady noise and babble	Improved 36 to 45% correct in steady noise; no improvement in babble (14 to 18%)	Yes, only in steady noise *		
Reis (2021) [41]	On test–text reception threshold (% of text obstructed)	Improvement ranged from 4% to 6% for various tasks	Yes *	1 to 3 months	Yes
	BKB/A sentences in +20SNR to 0SNR	No improvement at any time point post-training	No		
	CNC word in quiet	Improvement by 8% post training for nonvisual auditory training	Yes *		
	Spectral-temporally modulated ripple test	No improvement at any time point post-training	No		
	Speech, Sound, and Quality-12 questionnaire	No improvement at any time point post-training	No		
	Personal Report of Communication Apprehension	No improvement at any time point post-training	No		
	Self-efficacy for Situational Communication Management Questionnaire	No improvement at any time point post-training	No		
	Quality-of-Life Scale	Improvement of 5.1 out of 100 post-training for nonvisual auditory training	Yes *		
Reynard (2022) [40]	French sentence recognition-in-noise matrix test (dS SNR to reach 70% recognition)	SNR lower by −3.98 dB in the experiment group post-training and −2.28 at 5-week follow-up; no change in control; improvement not correlated with hours of training	Yes ***	5 weeks	No
	On test–speech reception threshold (dB SNR)	Improvement ranged from 1.26 dB to 4.13 dB for various tasks	Yes *		
Schumann (2015) [39]	Speech recognition in noise +5SNR (Goettingen Sentence Test)	Improvement by 10% in trained group immediately and 8.4% in 6 months; no change in control	Yes **	6 months	No
	Speech recognition in noise 0 SNR (Goettingen Sentence Test)	Improvement by 8% in trained group immediately and 7.3% in 6 months; no change in control	No		
Shafiro (2015) [36]	CNC word in quiet	Improvement of 4%	No	No long-term follow-up	Yes
	SPIN-R	Improvement of 3%	No		
Stacey (2010) [13]	Vowel recognition	Improvement of 3%	No	No long-term follow-up	No
	Consonant recognition	Improvement of 8%	Yes *		
	BKB sentences in quiet	No improvement of −0.25%	No		
	IEEE sentences in quiet	Improvement of 4%	No		
	Glasgow Benefit Inventory	Average improvement of 4.43 out of 100	No		
Tyler a (2010) [28]	Spondee in noise reception (dB SNR)	Improvement in two subjects—one from −4.8 to −7.8 SNR; other scores not in text	Yes **	No long-term follow-up	Yes
	CNC word in quiet	No improvement in any subject	No		
	CUNY sentences in noise	No improvement in any subject	No		
	HINT sentences in noise	Improvement in 2 subjects of 32% and 36%	Yes ***		
Tyler b (2010) [28]	Spondee word recognition with spatial cueing	Improvement in 2 subjects compared to controls, no values in text	Yes ***	7-month results for 1 subject	No
	Spondee word recognition with jammers from multiple locations	Improvement in 2 subjects compared to controls, no values in text	Yes **		
Völter (2021) [37]	Freiburg Speech Intelligibility Test	No improvement	No	No long-term follow-up	Yes
	Hochmair–Schulz–Moser Sentence Test	Improvement after CBAT training period	Yes **		
	Speech tracking	Improvement in rate at each portion of training from 31.3 words per minute to 41.3 by the end of the study	Yes **		
	Phoneme discrimination	Improvement seen in vowel and consonant discrimination after CBAT training	Yes *** and Yes *, respectively		
	Pseudoword identification	No improvement	No		
	Oldenburger Inventory Score	Improvement seen in the “listening in noise” subcategory after clinician-directed training	Yes **		
Zhang (2012) [38]	Vowel identification	Improvement of 9%	Yes *	1 month	No
	Consonant identification	Improvement of 10%	Yes *		
	CNC word in noise	Improvement of 15%	Yes *		
	AzBio in quiet in noise	Improvement of 8.3%	No		

* *p* < 0.05. ** *p* < 0.01. *** *p* < 0.001. BKB = Bamford–Kowel–Bench, CDT = clinician-directed training, CBAT = computer-based auditory training, CNC = consonant-nucleus-consonant, CUNY = City University of New York, IEEE = Institute of Electrical and Electronics Engineers, HINT = hearing in noise, MBAA = marginal benefit from acoustic amplification, SIN = speech in noise, SPATS = Speech Perception Assessment and Training System, SPIN-R = revised speech perception in noise test, and SRT = speech recognition threshold.

**Table 5:** Demographic summary of study samples included in meta-analysis.

Patient Factor	*n*	Patient CBAT	*n*	Clinician-Guided CBAT	*n*	Mixed CBAT
Mean age at intervention	82	60.6 ± 10.3 (25–87)	21	63.0 ± 13.1 (35–81)	29	63.9 ± 15.8 (26–84)
Sex, *n* (%)	47		13		29	
Male		17 (35.4)		4 (30.8)		12 (41.4)
Female		30 (64.6)		9 (69.2)		17 (58.6)
Duration of hearing loss in years, ***mean (SD, range)***	50	26.4 ± 22.5 (3–68)	21	20.4 ± 18.6 (2–61)	29	28.5 ± 17.8 (1–74)
Duration of CI experience in years, ***mean (SD, range)***	82	4.84 ± 4.18 (0.83–25.0)	21	1.35 ± 1.36 (0.25–5.0)	29	2.28 ± 5.67 (0.25–34.0)
Hearing aid use, *n* (%)	45	15 (33.3)	13	0 (0)	8	1 (12.5)

CBAT = computer-based auditory training; SD = standard deviation.

**Table 6:** Pooled mean difference of intervention by subtype.

CBAT	Speech Recognition Outcome	Mean Difference [95% CI]
**Patient-Directed**	Phoneme—Consonant	4.10 [−5.53, 13.73]
	Phoneme—Vowel *	8.01 [0.08, 15.94]
	Words in Quiet	3.24 [−5.11, 11.60]
	Sentence in Quiet	6.78 [−9.25, 22.81]
	Sentence in Noise *	10.33 [4.16, 16.49]
**Clinician-Directed**	Sentences in Noise *	21.80 [17.53, 26.07]
**Mixed**	Words in Quiet	5.37 [−7.78, 18.53]
	Sentence in Noise	6.79 [−9.81, 23.38]

CBAT = computer-based auditory training; CI = confidence interval. * Significant increase in mean difference post-intervention (*p* < 0.05).

**Table 7:** Study quality scores and levels of evidence for included articles.

Author	Year	Randomization	Control Group	Power Calculation/Sample Size	Blinding	Outcome Reporting	External Validity of Outcome Measure	External Validity of Training/Training Environment	Training Feedback	Long-Term Follow-Up	Reporting of Compliance	Total	Level of Evidence *
Barlow [29]	2016	0	0	0	0	2	2	2	2	0	2	10	Low
Berstein [45]	2021	2	2	2	1	2	2	0	1	1	2	15	Moderate
Borel [30]	2020	0	0	0	0	2	2	1	1	1	2	9	Low
Dornhoffer [21]	2022	0	1	1	0	2	2	2	0	1	0	9	Low
Gagne [31]	1991	0	0	0	0	0	1	1	0	0	2	4	Very low
Green [32]	2019	0	0	0	0	2	2	2	2	1	2	11	Moderate
Fu [20]	2004	0	0	0	0	2	2	1	2	0	0	7	Low
Ihler [44]	2017	2	0	0	0	1	2	2	0	0	2	9	Low
Ingvalson [33]	2013	0	0	0	0	0	2	0	0	1	0	3	Very Low
Kerneis [34]	2023	0	0	0	0	1	2	2	2	1	2	10	Low
Magits [43]	2023	2	2	1	2	1	2	1	2	1	2	16	Moderate
Miller [42]	2008	2	2	0	0	1	2	0	0	0	2	9	Low
Miller [46]	2016	0	1	0	0	2	1	0	2	0	2	8	Low
Moberly [47]	2020	0	1	0	0	2	2	2	2	0	1	10	Low
Oba [35]	2011	0	0	0	0	2	2	2	2	1	2	11	Moderate
Reis [41]	2021	2	2	1	0	2	2	1	2	1	2	15	Moderate
Reynard [40]	2022	2	2	1	0	2	2	1	2	1	2	15	Moderate
Schumann [39]	2015	2	2	0	0	2	2	0	2	2	0	12	Moderate
Shafiro [36]	2015	0	0	0	0	2	2	2	2	0	0	8	Low
Stacey [13]	2010	0	0	0	0	2	2	2	2	0	2	10	Low
Tyler a [28]	2010	0	1	0	0	1	1	1	2	1	0	7	Low
Tyler b [28]	2010	0	1	0	0	1	1	1	2	1	0	7	Low
Völter [37]	2021	0	0	0	0	1	2	1	2	0	0	6	Low
Zhang [38]	2012	0	0	0	0	2	2	2	2	1	2	11	Moderate

0—flawed or no information from which to make a judgement; 1—weak information or lack of detail; and 2—appropriate use and reporting. * Total score 0–5: very low level of evidence; 6–10: low level of evidence; 11–15: moderate level of evidence; and 16–20: high level of evidence.
